# Development of Photoresponsive Water-Soluble Superhydrophobic Coatings and Properties of the Modified Paper

**DOI:** 10.3390/polym17192615

**Published:** 2025-09-27

**Authors:** Shangjie Jiang, Yonghui Zuo

**Affiliations:** 1 School of Mechanical Engineering & Automation, University of Science and Technology Liaoning, An’shan 114051, China; 2 Innovative Team for the Development of Energy-Saving and Environmentally Friendly Metallurgical New Processes and Equipment, An’shan 114051, China

**Keywords:** superhydrophobic paper, water-soluble, light-responsive, wettability

## Abstract

In this study, a highly stable light-responsive superhydrophobic paper was successfully fabricated. The process involved polymerizing the synthesized light-responsive monomer PAPAE with the hydrophilic monomer 2-hydroxyethyl methacrylate(HEMA), the fluorine-containing monomer hexafluorobutyl methacrylate(HFMA),and 3-trimethoxysilyl-propyl methacrylate(TSPM), followed by grafting (3-Aminopropyl) triethoxysilane (APTES)-modified SiO_2_ nanoparticles onto the polymer to enhance surface roughness, and subsequently applying this composite to the paper surface. When the monomer ratio in the polymer was HFMA:TSPM:PAPAE:HEMA = 0.2:0.2:0.4:0.2, the resulting coating exhibited good water solubility, enabling the modified paper to achieve reversible wettability transitions under light irradiation. At a SiO_2_-to-polymer ratio of 0.3, the contact angle variation range reached its maximum (96–156.8°). The proposed method for fabricating superhydrophobic paper not only offers relative simplicity, low cost, and strong versatility but also imparts the paper with excellent weather resistance, abrasion resistance, and ultrasonic durability, highlighting its great potential for practical applications.

## 1. Introduction

Due to their unique wettability, superhydrophobic surfaces have found widespread applications in daily life. However, in recent years, increasing demands from industrial production and harsher application environments have revealed the limitations of single wettability properties, which often fail to meet practical requirements [1,2,3,4]. To address this challenge, researchers have explored diverse strategies for preparing functional superhydrophobic materials, including temperature-, light-, pH-, electric field-, and ionic liquid-responsive approaches [5,6,7,8,9,10,11]. These studies have laid a solid foundation for the further development and optimization of functional superhydrophobic papers. Among these approaches, light-responsive superhydrophobic papers have attracted particular interest because they allow remote, precise control of surface wettability and are relatively easy to apply in practical production [12].

The principle underlying light-responsiveness lies in the molecular isomerization of photosensitive groups on the material surface. Specifically, upon exposure to light of different wavelengths, these molecules undergo significant conformational changes, thereby inducing reversible transitions in surface wettability [13]. Nevertheless, current research on light-responsive superhydrophobic surfaces remains limited, and many proposed preparation methods are derived from those for other functional surfaces. These methods often rely on scarce or expensive materials and involve complex fabrication processes, which restrict their practical applicability [14,15,16]. For example, spiropyran-modified silicon surfaces achieve wettability switching but suffer from poor substrate adaptability and recyclability [17]. DNA-based self-assembled coatings exhibit pH responsiveness yet are costly and technically complex [18]. Similarly, a pH-responsive superhydrophobic surface prepared by grafting a block copolymer onto a protein-coated inverse opal film demonstrated weak adhesion between the polymer and rough substrate, resulting in poor stability [19].

The regulation of surface wettability is of great importance in functional material design. Classical theories such as the Wenzel and Cassie models describe the wetting behavior of rough surfaces: the Wenzel model corresponds to complete liquid infiltration into surface grooves (hydrophilic state), while the Cassie model describes composite contacts between droplets, solid asperities, and trapped air (superhydrophobic state). Building on these theories, light-responsive polymers can dynamically tune surface energy through molecular structural changes (e.g., azobenzene cis–trans isomerization), thereby enabling reversible switching between the Wenzel and Cassie states [20,21].

In this study, a light-responsive superhydrophobic polymer was synthesized via copolymerization of PAPAE, HFMA, TSPM, and HEMA. To further enhance surface roughness and stability, aminated SiO_2_ nanoparticles were grafted onto the polymer. The resulting composite material can be conveniently processed into powder for long-term storage and rapidly redissolved Ultraviolet lampin water for practical use. When applied to paper substrates, it forms a superhydrophobic coating that exhibits not only light responsiveness but also excellent stability, attributed to the incorporation of silica.

## 2. Experimental Section

### 2.1. Materials

The main materials and reagents used in this study are listed as follows. All chemicals were used as received without further purification. Wood pulp paper (Duni, Malmö, Sweden); sheet size: 40 cm × 40 cm; sheet weight: 9.5 g; thickness: 0.48 mm; surface smoothness: 87 s; 3-trimethoxysilyl propyl methacrylate (TSPM), 2-hydroxyethyl methacrylate (HEMA), hexafluorobutyl methacrylate (HFMA) (Sigma, St. Louis, MO, America); APTES (Union Silicon, Nanjin, China); nano-SiO_2_ 70 nm(Aladdin, Shanghai, China); 4-hydroxyazobenzene, potassium carbonate, potassium iodide, *n*-butanol, 2-chloroethanol, acryloyl chloride, ethyl chloride, sodium bisulfite (NaHSO_3_), tetrahydrofuran, aluminum oxide, potassium bromide (Aladdin, Shanghai, China); azobisisobutyronitrile (AIBN, Four Hevi, Shanghai, China); anhydrous ethanol (Miura, Matsuyama, Japan); and triethylamine (Tianli, Tianjin, China).

### 2.2. Preparation of the Light-Responsive Monomer PAPAE

**Figure 1:**
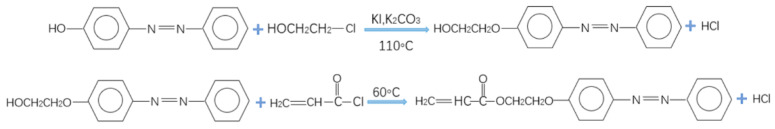
Synthetic route of light-responsive monomer PAPAE.

After 4-hydroxyethoxyazobenzene was prepared by reacting 4-hydroxyazobenzene with 2-chloroethanol, the PAPAE monomer can be prepared by reacting it with acryloyl chloride(Figure 1) [22]. First, 4-hydroxyethoxyazobenzene was obtained by reacting 4-hydroxyazobenzene with 2-chloroethanol. Specifically, 4-hydroxyazobenzene (19.8 g, 0.1 mol), potassium carbonate (13.8 g, 0.1 mol), and potassium iodide (0.6 g, 3.6 mmol) were dissolved in 60 mL of *n*-butanol under a nitrogen atmosphere. After stirring, 2-chloroethanol (6.75 mL, 0.1 mol) was added, and the mixture was heated in an oil bath at 110 °C for 7 h. The resulting product was centrifuged at 3000 rpm for 30 min, washed with absolute ethanol, and dried to yield 4-hydroxyethoxyazobenzene.

In the second step, 4-hydroxyethoxyazobenzene (6 g, 0.025 mol) and triethylamine (3 g, 0.025 mol) were added dropwise to a solution of acryloyl chloride (2.24 g, 0.025 mol) in 100 mL of ethyl chloride. The mixture was placed in a round-bottom flask, sealed, and frozen in liquid nitrogen. After complete freezing, the solution was evacuated, flushed with nitrogen, and thawed; this freeze–pump–thaw cycle was repeated twice. The reaction mixture was then heated to 60 °C and stirred overnight under a nitrogen atmosphere. The product was collected by centrifugation at 3000 rpm for 30 min, washed with absolute ethanol, and dried, yielding the target light-responsive monomer PAPAE.

### 2.3. Preparation of Water-Soluble Light-Responsive Polymer

**Figure 2:**
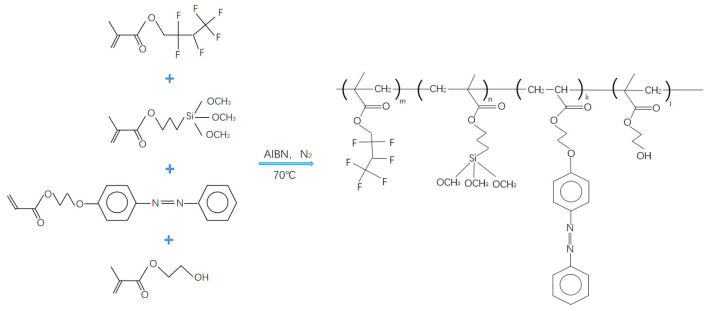
Synthetic route of water-soluble light-responsive polymer.

The water-soluble light-responsive polymer was synthesized through the following steps (Figure 2). First, TSPM was purified by passing it through an alumina chromatography column to remove polymerization inhibitors. Second, HFMA, TSPM, PAPAE, and HEMA were placed in a round-bottom flask, to which 100 mL of tetrahydrofuran (THF) and AIBN (0.002 mol, 0.33 g) were added. The flask was sealed, frozen in liquid nitrogen, and subjected to freeze–pump–thaw cycles (three repetitions) to remove dissolved oxygen. Third, the flask was transferred to a water bath maintained at 70 °C, and the mixture was magnetically stirred under a nitrogen atmosphere for 5 h. The resulting product was filtered twice and dried in an oven to yield a light-yellow transparent solid, designated PHFMA-PTSPM-PAPAE-PHEMA.

### 2.4. Preparation of Amino Modified SiO_2_

Amino-modified SiO_2_ nanoparticles were synthesized in four main steps. First, nano-SiO_2_ (1 g) was ultrasonically dispersed in ethanol (50 mL) for 1 h to obtain a uniform Si–ethanol suspension. Triethylamine (20 g) was then added dropwise to adjust the dispersion to an alkaline environment. Second, the mixture was transferred into a three-necked flask equipped with a reflux condenser, and the coupling agent APTES (1 g) was introduced. The reaction was conducted in a water bath at 75 °C under magnetic stirring for 12 h. Third, the product was purified by three cycles of centrifugation (3000 rpm, 45 min), washing with absolute ethanol, and redispersion. Finally, the purified precipitate was dried to yield amino-modified SiO_2_ nanoparticles.

### 2.5. Preparation of Light-Responsive Superhydrophobic Paper

**Figure 3:**
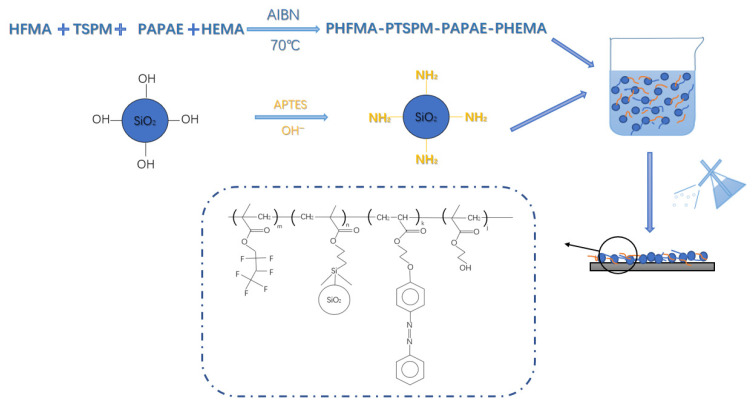
Preparation procedure of light-responsive superhydrophobic paper.

The preparation of light-responsive superhydrophobic paper is illustrated in Figure 3. First, the amino-modified SiO_2_ powder was dispersed in ethanol to form a homogeneous suspension. Separately, the synthesized water-soluble light-responsive polymer PHFMA-PTSPM-PAPAE-PHEMA (10 g) was dissolved in 50 mL of water. The SiO_2_–ethanol suspension was then added to the polymer solution and magnetically stirred for 1 h, yielding a water-soluble light-responsive superhydrophobic coating. Finally, the coating was sprayed uniformly onto the paper surface using an HD-150 spray gun (spray height: 15 cm, spray pressure: 0.15 MPa), followed by drying at 70 °C. Each milliliter of coating solution could cover an area of approximately 5 cm × 5 cm.

### 2.6. Characterization

The wettability of the samples was characterized using a contact angle goniometer DSA100 (KRUSS, Shanghai, China). A 5 μL droplet of deionized water was used for each measurement, and the contact angle and sliding angle were recorded at 10 randomly selected positions, with the average value reported. Fourier Transform Infrared Spectroscopy (FT-IR) analysis was performed using a Bruker VECTOR-22 (BRUKER, Beijing, China) spectrometer. X-ray Photoelectron Spectroscopy (XPS) was carried out on an Axis Ultra (KRATOS, Manchester, UK) spectrometer with Al Kα radiation (1486.71 eV) under conditions of 10 mA current and 10 kV voltage. Thermogravimetric analysis (TGA) was conducted with a STA449C (NETZSCH, Selb, Germany) TG analyzer in an air atmosphere at a heating rate of 10 °C/min. The photo-induced experiments were performed using a 365 nm UV lamp (MOTIE, Tianjin, China) as the ultraviolet source and a 380–780 nm xenon lamp (ZOLIX, Beijing, China) as the visible light source. The samples were alternately exposed to UV light and visible light for 5 cycles, with each cycle consisting of 30 min of UV irradiation followed by 30 min of visible light irradiation.

Weather resistance was evaluated by exposing the modified paper to outdoor conditions, and the contact angle was measured at five random positions at different exposure times, with the average reported. Abrasion resistance was tested by attaching sandpaper to the base of a 500 g weight and rubbing it back and forth along a horizontal direction at 2 cm/s. Each 20 cm of friction distance was defined as one wear cycle. Contact angles were measured at five random positions after different cycles. Ultrasonic resistance was tested by immersing the modified paper in 100 mL of deionized water and subjecting it to ultrasonic treatment. At different treatment times, the paper was removed, dried at 80 °C, and the contact angle was measured at five random positions, with the average recorded.

## 3. Results and Discussion

### 3.1. Infrared Characterization of Polymers

**Figure 4:**
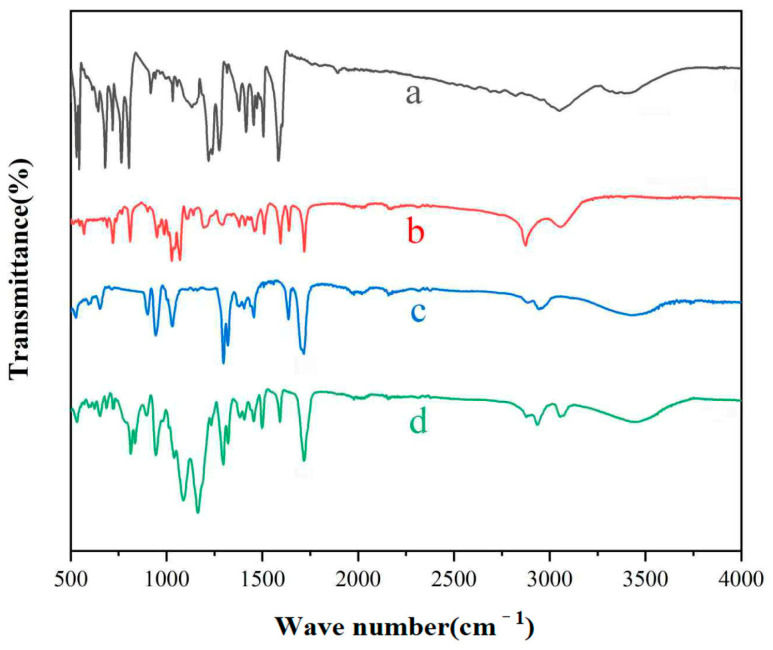
Fourier infrared spectrum of (**a**) 4-hydroxyazobenzene; (**b**) PAPAE; (**c**) HEMA; (**d**) PHFMA-PTSPM-PAPAE-PHEMA.

Figure 4 shows the infrared spectra of tetrahydroxyazobenzene, light-responsive monomer PAPAE, water-soluble monomer HEMA, and water-soluble light-responsive polymer PHFMA- PTSPM-PAPAE-PHEMA. Figure 4a is the infrared spectrum of tetrahydroxyazobenzene. As can be seen from the figure, 3075 cm^−1^ is the characteristic absorption peak of C-H on the aromatic ring, 3455 cm^−1^ is the stretching vibration peak of hydroxyl O-H, 1591 cm^−1^ is the stretching vibration peak of nitrogen double bond, 1501 cm^−1^ is the stretching vibration peak of C=C on the benzene ring skeleton, and 795 cm^−1^ is the out of plane bending vibration peak of benzene ring C-H. Figure 4b is the infrared spectrum of the light-responsive monomer PAPAE. In addition to the characteristic peaks of Figure 4a, the C=C stretching vibration peak appeared at 1635 cm^−1^, C-H methylene stretching vibration peak appeared at 2876 cm^−1^, C=O stretching vibration absorption peak appeared at 1721 cm^−1^, and hydroxyl O-H stretching vibration peak disappeared at 3462 cm^−1^. These changes in the infrared spectrum indicate the synthesis of the light-responsive monomer PAPAE. Figure 4c is the infrared spectrum of the water-soluble monomer HEMA. As can be seen from the figure, 3465 cm^−1^ is the stretching vibration peak of hydroxyl O-H, 2876 cm^−1^ is the stretching vibration peak of methylene, 1719 cm^−1^ is the C=O stretching vibration absorption peak, and 1635 cm^−1^ is the C=C stretching vibration peak. Figure 4d is the infrared spectrum of the water-soluble light-responsive polymer PHFMA-PTSPM-PAPAE-PHEMA. As can be seen from the figure, 3075 cm^−1^ is the characteristic peak of CH on the aromatic ring, 1595 cm^−1^ is the stretching vibration peak of the nitrogen-nitrogen double bond, 1500 cm^−1^ is the C=C stretching vibration peak on the benzene ring skeleton, 791^−1^ is the out-of-plane bending vibration peak of the benzene ring C-H, these peaks are all characteristic peaks of PAPAE; in addition, 3460 cm^−1^ is the stretching vibration absorption peak of hydroxyl O-H, 2876 cm^−1^ and 3957 cm^−1^ are the stretching vibration peaks of C-H methylene and methyl, 1720 cm^−1^ is the stretching vibration peak of C=O, 837 cm^−1^ is the symmetrical stretching vibration peak of Si-C, 1088 cm^−1^ is the stretching vibration peak of Si-O-C, 1166 cm^−1^ is the stretching vibration absorption peak of C-F, and the stretching vibration peak of C=C at 1635^−1^ disappears. As shown in Figure 4, the characteristic peaks observed in Figure 4b,c are also discernible in the infrared spectrum of the light-responsive polymer HFMA-TSPM-PAPAE-HEMA. This spectral consistency provides compelling evidence for the successful synthesis of the polymer and unequivocally confirms the effective incorporation of the designed monomer units into the polymer backbone [23,24].

### 3.2. XPS Test on Different Papers

**Figure 5:**
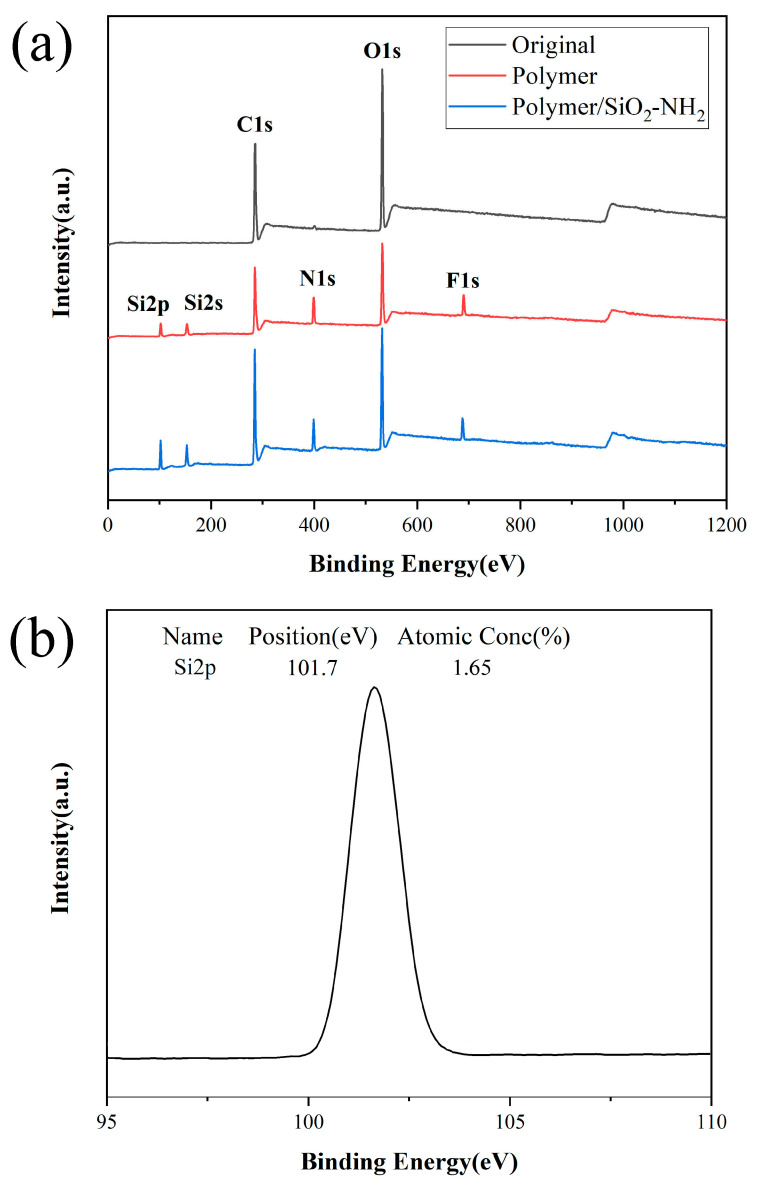

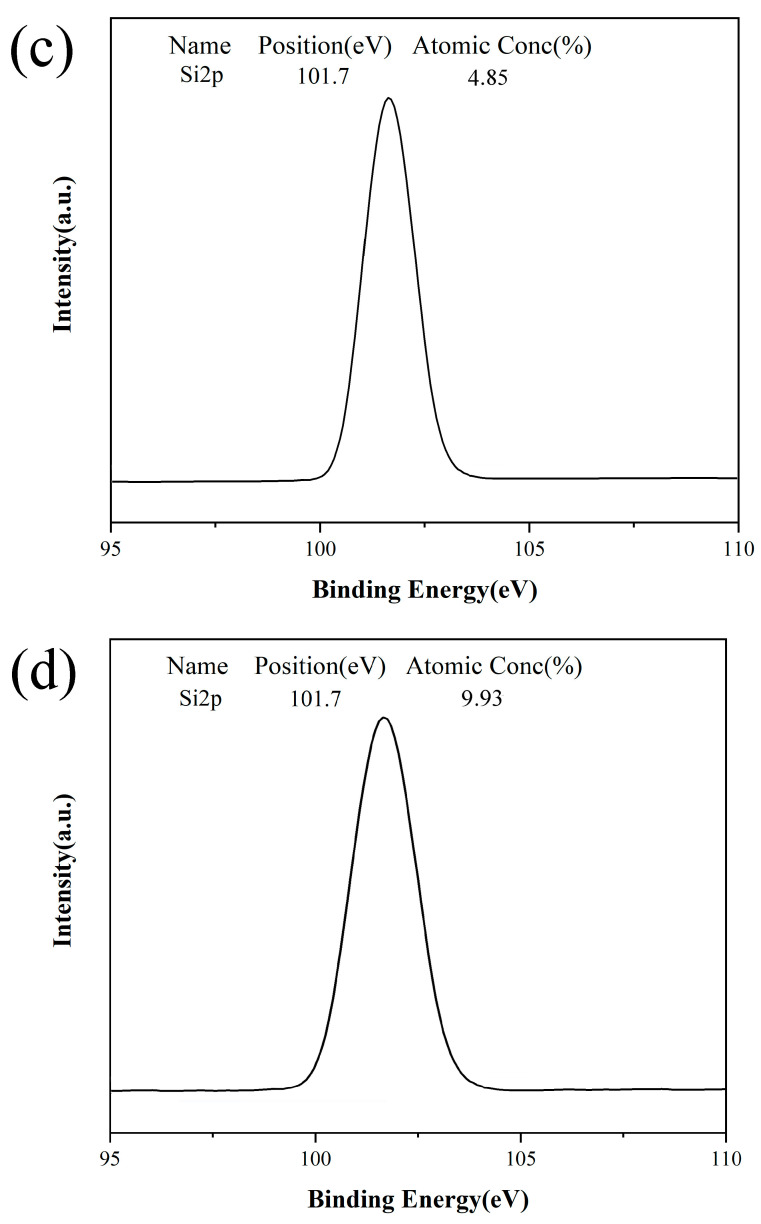
(**a**) XPS spectra. Si2p core level of (**b**) Original paper; (**c**) Paper coated with polymer PHFMA-PTSPM-PAPAE-PHEMA; (**d**) Paper coated with superhydrophobic coating PHFMA-PTSPM-PAPAE-PHEMA/SiO_2_-NH_2_.

Figure 5 shows the results of XPS analysis of original paper, PHFMA-PTSPM-PAPAE-PHEMA coated paper, and PHFMA-PTSPM-PAPAE-PHEMA/SiO_2_-NH_2_ coated superhydrophobic paper, in order to reveal the qualitative information of the chemical changes on the surface of the paper before and after the modification. Figure 5a shows the XPS spectra of the three papers. It can be seen from Figure 5 that the surface of the original paper is mainly composed of C and O elements, corresponding to the positions near 285 eV and 535 eV, respectively. Figure 5c is the Si2p spectrum of the paper after coating the polymer PHFMA-PTSPM-PAPAE-PHEMA. Compared with the Si2p spectrum of the original paper (Figure 5b), the surface of the paper coated with polymer has four peaks at 101 eV, 151 eV, 396 eV and 689 eV, corresponding to the appearance of Si2p, Si2s, N1s, and F1s signals, this shows that the polymer PHFMA-PTSPM-PAPAE-PHEMA was successfully modified on the paper surface. Figure 5d is the Si2p spectrum of the paper after coating the superhydrophobic coating PHFMA-PTSPM-PAPAE-PHEMA/SiO_2_-NH_2_. Compared with the original paper and the paper coated with the polymer PHFMA-PTSPM-PAPAE-PHEMA, the Si peak signal on the surface of the paper is stronger, which is mainly caused by the addition of SiO_2_, which also shows that the superhydrophobic coating PHFMA-PTSPM-PAPAE- PHEMA/SiO_2_-NH_2_ was successfully compounded with the paper surface [25].

### 3.3. Effect of Monomer Addition on the Properties of Modified Paper

To evaluate the influence of the light-responsive monomer PAPAE on the wettability of the modified paper, amino-modified SiO_2_ was grafted onto copolymers with different PAPAE ratios to prepare light-responsive superhydrophobic paper. The contact angles were then measured to identify the optimal monomer ratio. Table 1 summarizes the contact angles and light-responsive behavior of the modified papers prepared under different monomer compositions.

When the monomer ratio was HFMA:TSPM:PAPAE:HEMA = 0.2:0.2:0.1:0.2, the resulting paper exhibited neither superhydrophobicity nor light-responsiveness. Upon increasing the PAPAE content to 0.2, the prepared paper showed light-responsive behavior with a contact angle of 140.6° under hydrophobic conditions. Further increasing the PAPAE proportion to 0.4 yielded a contact angle of 156.6°, achieving both superhydrophobicity and light-responsiveness. When the PAPAE ratio was further raised to 0.6, the paper maintained its light-responsive property, and the contact angle was 156.8°.

These results indicate that once the PAPAE content exceeds a certain threshold, further addition neither increases the contact angle nor improves the light-responsiveness of the modified paper. Considering both preparation cost and the simultaneous attainment of superhydrophobicity and light-responsiveness, the optimal monomer composition was determined to be HFMA:TSPM:PAPAE:HEMA = 0.2:0.2:0.4:0.2. At this ratio, the modified paper exhibited the desired properties most effectively.

**Figure 6:**
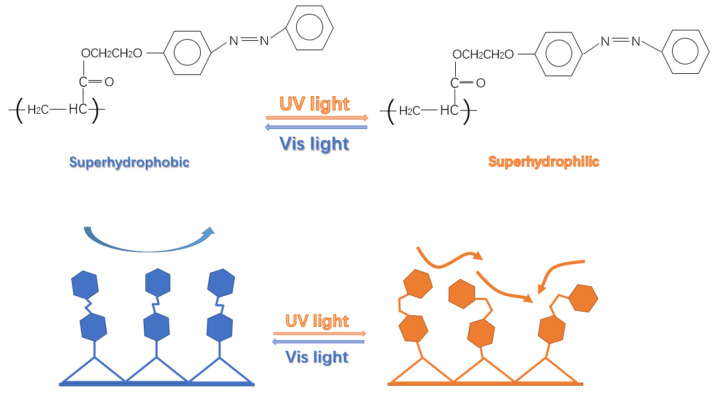
Analysis of the light-responsive mechanism of PAPAE.

Two modified paper samples (Sample 2 and Sample 3), prepared with different ratios of light-responsive monomers, were selected to evaluate the changes in surface contact angle before and after light irradiation, as well as their reversibility. As shown in Figure 6, when Sample 3 was irradiated with ultraviolet (UV) light for 30 min, its contact angle decreased sharply from 156.6° to 92°. Subsequent irradiation with visible light for 30 min restored its original superhydrophobicity, demonstrating that the wettability of the light-responsive superhydrophobic paper is reversible.

This reversible wettability originates from the unique photoresponsive properties of the azobenzene structure, a typical light-sensitive organic unit. Under UV irradiation, azobenzene undergoes a trans-to-cis isomerization, which increases the molecular dipole moment and raises the surface energy, thereby reducing the contact angle. In contrast, exposure to visible light induces the reverse cis-to-trans transformation, lowering the dipole moment and surface energy, which restores a higher contact angle and the superhydrophobic state [26].

**Figure 7:**
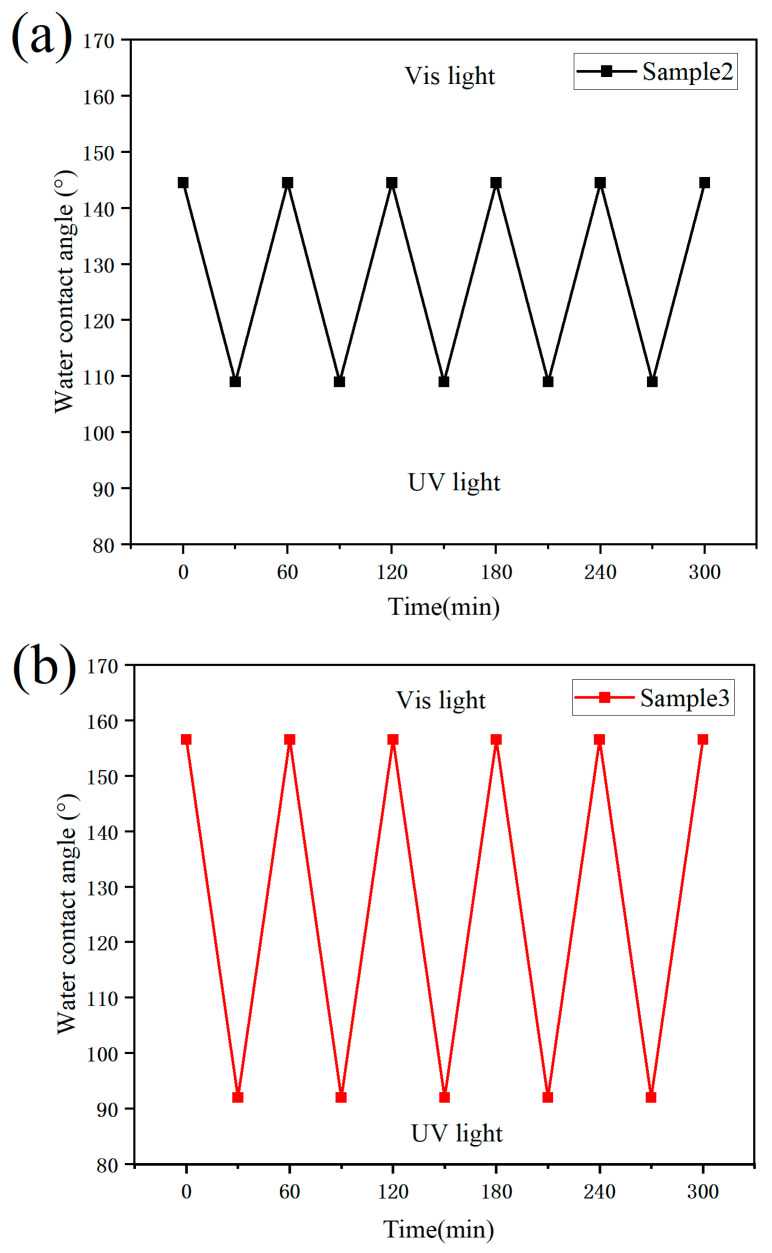
Variation of contact angle of modified paper under light induction: (**a**) Sample 2 and (**b**) Sample 3.

Figure 7 shows the variations in contact angle of Samples 2 and 3 during five successive cycles of alternating visible and UV light irradiation, with each cycle lasting 30 min. As illustrated, the range of contact angle change in Sample 3 was greater than that of Sample 2. This indicates that, within a certain range, the degree of light-induced wettability change is positively correlated with the content of light-responsive monomers in the polymer.

From a practical perspective, however, the contact angle variations in the light-responsive superhydrophobic paper did not span the full range from superhydrophilicity to superhydrophobicity. Instead, the transition occurred only between hydrophobic and superhydrophobic states. This limitation can be attributed to the short distance between the polymer backbone and the azobenzene moieties, which increases steric hindrance and ring-opening resistance among adjacent azobenzene units. Consequently, the conformational transition of some azobenzene molecules is restricted, reducing their effective interaction with water molecules after UV irradiation [27].

To investigate the influence of the content of the water-soluble monomer HEMA on the surface wettability of modified paper and the water solubility of the coating, poly-mers with varying HEMA ratios were synthesized and grafted onto amino-modified SiO_2_ to prepare light-responsive superhydrophobic paper. The water solubility of the coatings and the surface contact angle of the modified paper were subsequently ana-lyzed. Table 2 presents the contact angles and water solubility of the modified paper surfaces prepared with different monomer ratios. 

**Figure 8:**
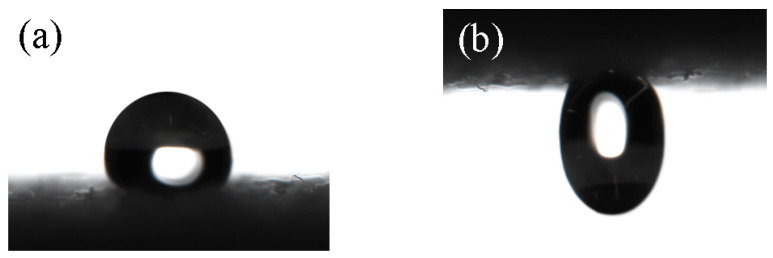
The superhydrophobic paper showed the strongest surface adhesion at a monomer ratio of HFMA:TSPM:PAPAE:HEMA = 0.2:0.2:0.4:0.3. (**a**) Place water droplets on a horizontal surface. (**b**) Test the adhesion force on levitated water droplets.

As shown in Table 2, when the monomer ratio was HFMA:TSPM:PAPAE:HEMA = 0.2:0.2:0.4:0.1, the resulting coating was difficult to dissolve in water, indicating negligi-ble water solubility. When the ratio was adjusted to HFMA:TSPM:PAPAE:HEMA = 0.2:0.2:0.4:0.2, the modified paper exhibited both excellent water solubility and super-hydrophobicity, with a surface contact angle reaching 156.3°. However, increasing the HEMA content further to HFMA:TSPM:PAPAE:HEMA = 0.2:0.2:0.4:0.3 resulted in a coating that, although still water-soluble, displayed a reduced contact angle of 139° and lost its superhydrophobic properties. At this stage, as illustrated in Figure 8, the paper surface showed strong adhesion to water droplets. This adhesion primarily arises from polar interactions between the hydrophilic HEMA groups in the polymer and water molecules, which induce a certain degree of droplet retention. Additionally, water droplets could partially penetrate the grooves on the paper surface, forming a “wet contact” state that further hindered droplet rolling.

From these observations, it can be concluded that an insufficient proportion of HEMA results in coatings that are not water-soluble, while an excessive HEMA content diminishes the hydrophobicity and increases droplet adhesion. Therefore, considering both the water solubility of the coating and the attainment of superhydrophobicity, the optimal monomer ratio was determined to be HFMA:TSPM:PAPAE:HEMA = 0.2:0.2:0.4:0.2, yielding modified paper with the most favorable combination of proper-ties.

### 3.4. Effect of Paper Surface Roughness on Light-Induced Wettability

**Figure 9:**
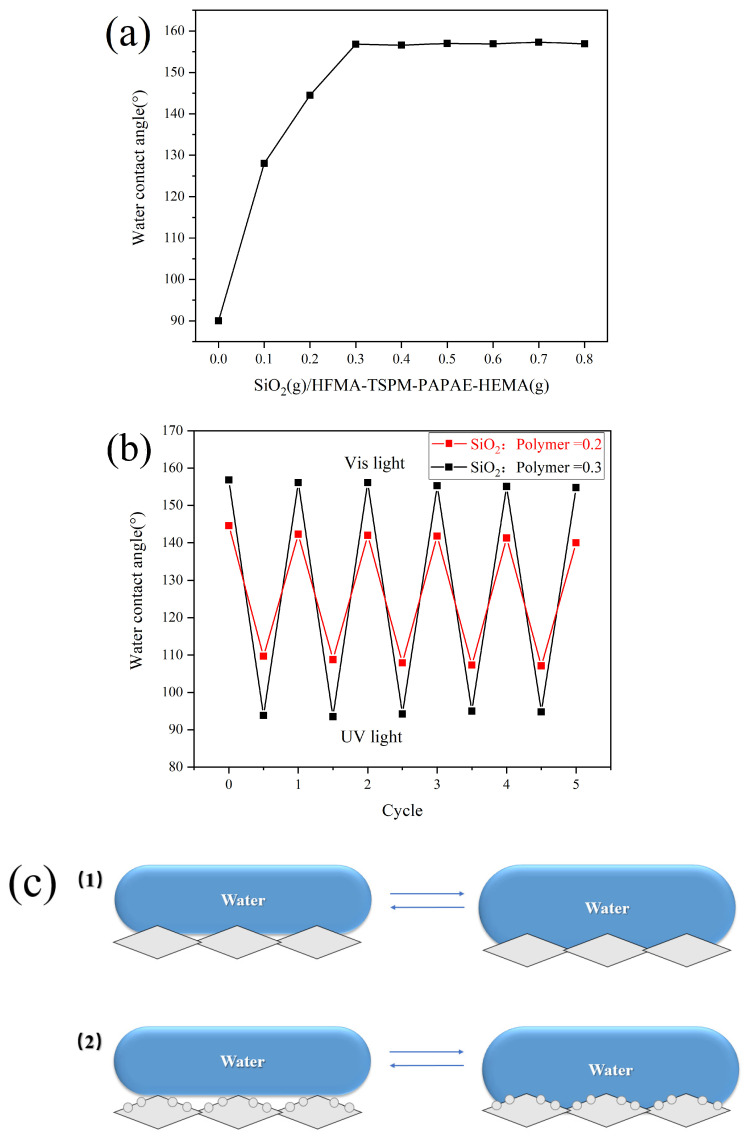
(**a**) The relationship between the mass ratio of SiO_2_ to polymer and the contact angle of paper surface; (**b**) The reversibility of the surface wettability of modified paper prepared under different mass ratios of SiO_2_ and polymer under UV-visible light induction; (**c**) The change in the light response wettability of the paper surface under different mass ratios of SiO_2_ to polymer: (**1**) 0.2, (**2**) 0.3.

In order to test the effect of the paper surface roughness on its light-induced wettability, the polymer was prepared when the monomer ratio was HFMA:TSPM:PAPAE:HEMA = 0.2:0.2:0.4:0.2, and the superhydrophobic and light-induced reversibility of the modified paper were tested under the conditions of different mass ratio of modified SiO_2_ to polymer. Figure 9a shows the change in the contact angle of the paper surface as the mass ratio of SiO_2_ to polymer increases. As can be seen from the figure, when the ratio of SiO_2_ content to polymer was 0.2, the paper had not yet obtained superhydrophobic properties due to the insufficient surface roughness (contact angle was 144.5°); When the ratio of SiO_2_ to polymer reached 0.3, the surface of the paper obtained sufficient roughness, so that the paper also obtained superhydrophobic properties (the contact angle reached 156.8°). However, as the SiO_2_ content continued to increase, the contact angle of the paper surface hardly changed. At this time, the excessive SiO_2_ content will not only not increased the surface contact angle, but also affected the transparency of the paper and other properties. Therefore, when the content ratio of SiO_2_ to polymer was 0.3, the properties of the prepared coating reached the best state.

Figure 9b shows the reversible conversion of the surface wettability of modified paper prepared under different SiO_2_ content ratios under the induction of ultraviolet-visible light. When the ratio of SiO_2_ to polymer content was 0.3, the contact angle of the paper surface was reduced from 156.8° to 96° after being irradiated with ultraviolet light, and the superhydrophobic property was lost. However, after being irradiated with visible light, the surface of the paper recovered its original superhydrophobic properties. The principle analysis of paper surface roughness on its light-induced wettability transition is as follows:

Based on the Cassie wetting model, the following theoretical Equation (1) describes the relationship between surface wettability and material roughness.

cos θ_cB_ = f_1_ cos θ_s_ − f_2_ (1)

In the equation:

f_1_—The percentage of the contact area between the droplet and the solid surface;

f_2_—The percentage of the contact area between the droplet and the air;

θ_s_—The static contact angle of the drop on the smooth surface;

θ_cB_—The static contact angle of a droplet on a rough surface.

In the study, the measured contact angle of the droplets on the smooth surface was 92°, the contact angle on the surface of the paper prepared under the condition of SiO_2_ content ratio of 0.2 was 144.5°, and the contact angle on the surface of the paper after ultraviolet light irradiation was 109°. According to Equation (1), when the ratio of SiO_2_ content to polymer was 0.2, the percentages of the area of the modified paper surface in contact with the droplets before and after ultraviolet light irradiation were 0.19% and 0.70%, respectively. Using the same method, we could see that when the ratio of SiO_2_ content to polymer was 0.3, the surface contact angle of the prepared modified paper was 156.8°, and the surface contact angle after ultraviolet light irradiation was 96°. Using Equation (1), it can be calculated that the percentages of the contact area between the modified paper surface and droplets before and after ultraviolet irradiation were 0.08% and 0.93%, respectively. It can be seen that the actual contact area between the water droplet and the paper surface will increase significantly after being irradiated by ultraviolet light, prompting the water contact mode of the material surface to gradually change to the Wenzel model. In the study, for the modified paper with SiO_2_ content to polymer at the ratios of 0.2 and 0.3, respectively, after continuous alternating irradiation with ultraviolet light and visible light, it was found that the change in surface contact angle was reversible and the change range of contact angle was relatively stable (Figure 9b). It can be inferred that the material surface roughness was an important factor affecting the reversible CIS-TRANS conformational transition range of light-induced azobenzene, when the chemical composition of the material surface was certain, the larger the surface roughness, the larger the range of light-induced reversible change.

Figure 9c shows the schematic diagram of the contact between the liquid droplet and the solid surface under the conditions of SiO_2_: Polymer = 0.2 and SiO_2_: Polymer = 0.3, the two fiber surfaces were all transformed into the Wenzel model state after being irradiated with ultraviolet light. It can be seen from the figure that when the surface of the fiber membrane was irradiated with ultraviolet light, the contact area between the paper surface and the droplet changed less when the SiO_2_ content ratio was 0.2. However, the roughness increases with the increased of the SiO_2_ content on the surface of the substrate, until the SiO_2_ content ratio reached 0.3, the contact area between the paper surface and the droplet changed more. The experimental results have been confirmed in the previous calculations, the contact area (36.4%) of the paper surface with the droplet under the condition of SiO_2_ content ratio of 0.3 was larger than the contact area (30.6%) under the SiO_2_ content ratio of 0.2 after ultraviolet light irradiation. According to the transformation Equation (2) of Equation (1):

cos θ_c_ = f_s_(cos θ + 1) − 1 (2)

In the equation:

θ—The static contact angle of the drop on the smooth surface;

θ_c_—The actual contact angle of the drop on the rough surface;

f_s_—The ratio of the contact area of the solid surface to the droplet.

According to Equation (2), when the cosθ remained unchanged, the contact area f_s_ between the solid surface and the droplet increased, the cos θ_c_ also increased while the obtained θ_c_ became smaller. Therefore, after ultraviolet light irradiation, the actual contact area ratio between the paper surface and water droplets prepared when SiO_2_: polymer = 0.3 was larger, and the contact angle was smaller than that when SiO_2_: polymer = 0.2. However, when the paper was irradiated by visible light, because there were more grooves for collecting air on the paper surface prepared when SiO_2_: polymer = 0.3, the real contact area f_s_ between the paper fiber surface and droplets was smaller than that prepared when SiO_2_: polymer = 0.2, and the paper fiber surface obtained a larger contact angle. Based on the above analysis, it can be seen that within a certain range, as the content of SiO_2_ on the surface of the substrate increases, the range of the reversible transformation of the contact angle of the light-induced substrate surface becomes larger.

### 3.5. Thermogravimetric Analysis

**Figure 10:**
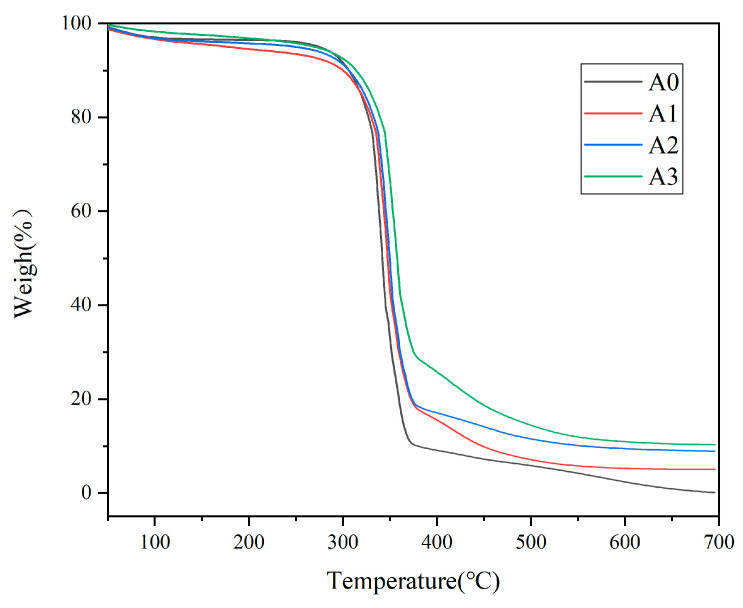
TGA curves of A0, A1, A2, A3, Samples were heated to 700 °C in air atmosphere at a ramp rate of 10 °C/min^−1^.

Figure 10 presents the thermogravimetric analysis (TGA) curves of paper samples A0, A1, A2, and A3. After heating all samples to 700 °C, the unmodified original paper sample A0 showed a residual weight of 0%, indicating complete combustion. For sample A1, which was coated with conventional SiO_2_ dispersion, the residual weight was 5.35%, primarily attributable to the remaining SiO_2_. Sample A2, prepared by spraying amino-modified SiO_2_, exhibited a residual weight of 9.63%. This increase is due to the reaction of APTES with hydroxyl groups on the paper fibers, with partial combustion of the grafted groups incorporated into the paper, indicating successful grafting of APTES onto the SiO_2_ surface. Sample A3, representing the light-responsive superhydrophobic paper modified with PHFMA-PTSPM-PAPAE-PHEMA/SiO_2_-NH_2_, had a residual weight of 10.6%, suggesting that the polymer composite on the surface of the amino-modified SiO_2_ particles enhanced the thermal stability of the material [28,29].

### 3.6. Stability Test

**Figure 11:**
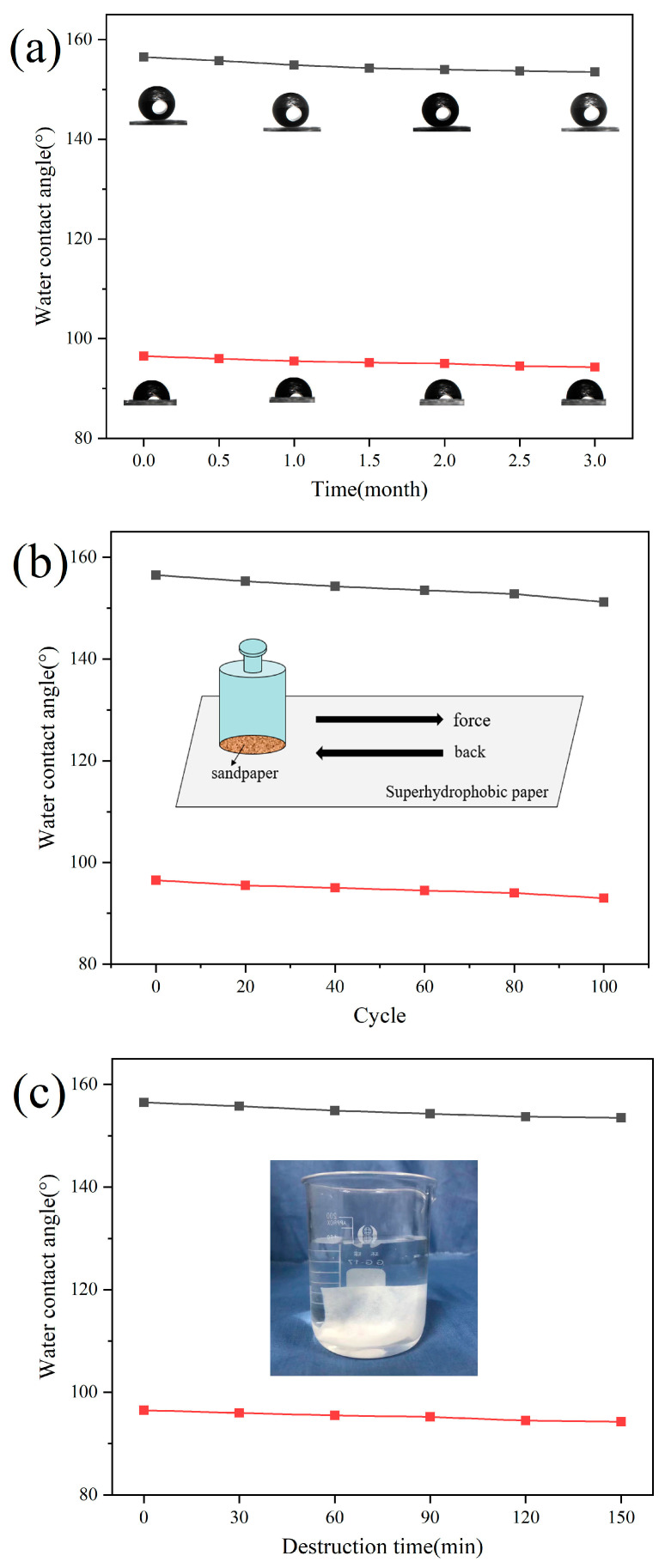
Changes in contact angle of modified paper before and after being destroyed: (**a**) exposure time for 3 months; (**b**) 100 friction cycles; (**c**) ultrasonic dispersion for 150 min.

Figure 11 illustrates the changes in contact angle of the modified paper under various destructive conditions, revealing the stability of its superhydrophobic properties. The effects of outdoor exposure for 3 months, 100 friction cycles, and 150 min of ultrasonic treatment on the contact angle under visible and ultraviolet light irradiation were evaluated.

Figure 11a shows the influence of outdoor exposure on hydrophobicity. Even after 3 months, the contact angles under visible and ultraviolet light decreased only to 153.5° and 93.8°, respectively, indicating minimal fluctuation. Figure 11b depicts the effect of abrasion resistance: after 100 rubbing cycles, the contact angles were slightly reduced to 151.2° and 93.8°, with the paper still maintaining superhydrophobic and hydrophobic states. Figure 11c illustrates the modified superhydrophobic paper sample immersed in a beaker containing deionized water and subjected to ultrasonic treatment. Notably, the contact angle remained essentially unchanged even after 150 min of ultrasonication, thereby providing strong evidence of the material’s remarkable stability.

Although prolonged ultrasonic exposure in industrial applications, such as oil–water separation, may slightly reduce hydrophobicity due to detachment of polymer and modified silica nanoparticles, the overall structural integrity and superhydrophobicity are preserved. These results further confirm that the microstructure of the modified paper is a critical factor in maintaining superhydrophobic performance.

## 4. Conclusions

In this study, a one-step method was employed to synthesize a water-soluble, light-responsive polymer from the monomers PAPAE (light-responsive), HEMA (hydrophilic), HFMA (fluorinated), and TSPM. Subsequently, APTES-modified silica was grafted onto the polymer via its surface amino groups to enhance surface roughness. When applied to paper, this nanocomposite coating formed a highly stable superhydrophobic surface capable of reversible wettability switching under alternating UV and visible light irradiation. The effects of varying monomer ratios on the water solubility of the superhydrophobic coating, as well as the surface wettability and light responsiveness of the modified paper, were systematically investigated. Considering both preparation cost and the achievement of optimal superhydrophobicity, the monomer ratio HFMA:TSPM:PAPAE:HEMA = 0.2:0.2:0.4:0.2 was determined to yield the best results. At this composition, the coating exhibited excellent water solubility, while the modified paper demonstrated strong light responsiveness, superhydrophobicity, and a wide range of reversible contact angle changes. Further investigation into the effect of paper surface roughness revealed that a SiO_2_-to-polymer ratio of 0.3 produced the largest reversible range of contact angle changes under light-induced conditions.

In summary, the superhydrophobic paper prepared via the present method offers a simple and cost-effective fabrication process with broad applicability. The material also exhibits excellent weather resistance, abrasion resistance, and ultrasonic stability, demonstrating significant practical value. Potential applications include moisture-proof packaging and industrial filter membranes for oil–water separation, highlighting its promising prospects for industrial and everyday use.

## Figures and Tables

**Table 1:** Surface contact angle and light-responsiveness of modified paper prepared under different monomer ratios.

	HFMA	TSPM	PAPAE	HEMA	Water Contact Angle	Responsiveness
**Sample 1**	0.2	0.2	0.1	0.2	135.5 ± 2.8°	×
**Sample 2**	0.2	0.2	0.2	0.2	140.6 ± 1.9°	√
**Sample 3**	0.2	0.2	0.4	0.2	156.6 ± 2.1°	√
**Sample 4**	0.2	0.2	0.6	0.2	156.8 ± 2.3°	√

**Table 2:** Surface contact angle and water solubility of modified paper prepared under different monomer ratios.

	HFMA	TSPM	PAPAE	HEMA	Water Contact Angle	Water Solubility
**Sample 1**	0.2	0.2	0.4	0.1	158.5 ± 1.6°	×
**Sample 2**	0.2	0.2	0.4	0.2	156.3 ± 1.7°	√
**Sample 4**	0.2	0.2	0.4	0.3	139 ± 2.6°	√

## Data Availability

The data that support the findings of this study are available from the corresponding author upon reasonable request.
